# Withdrawn: MicroRNA‐142‐5p inhibits autophagy in cardiomyocytes in a mouse model of hypoxia/reoxygenation

**DOI:** 10.1002/2211-5463.12879

**Published:** 2021-11-29

**Withdrawn**: ‘MicroRNA‐142‐5p inhibits autophagy in cardiomyocytes in a mouse model of hypoxia/reoxygenation’, Peng Huang, Qiaoyan Lin, Xiaofang Liu. The above article, published online on 11 May 2020 in Wiley Online Library (wileyonlinelibrary.com) as an Accepted Article, has been withdrawn by agreement between the journal Editor‐in‐Chief Miguel De la Rosa, the FEBS Press Editorial Office, and John Wiley and Sons, Ltd. The withdrawal has been agreed because the authors are not responding to requests to finalize their article for publication in the journal as the Version of Record. https://doi.org/10.1002/2211‐5463.12879

